# Breakfast Protein Source Does Not Influence Postprandial Appetite Response and Food Intake in Normal Weight and Overweight Young Women

**DOI:** 10.1155/2016/6265789

**Published:** 2016-01-17

**Authors:** Christina M. Crowder, Brianna L. Neumann, Jamie I. Baum

**Affiliations:** Department of Food Science, University of Arkansas, 2650 North Young Avenue, Fayetteville, AR 72704, USA

## Abstract

Breakfasts higher in protein lead to a greater reduction in hunger compared to breakfasts higher in carbohydrate. However, few studies have examined the impact of higher protein breakfasts with differing protein sources. Our objective was to determine if protein source (animal protein (AP) versus plant protein (PP)) influences postprandial metabolic response in participants consuming a high protein breakfast (~30% energy from protein). Normal weight (NW; *n* = 12) and overweight women (OW; *n* = 8) aging 18–36 were recruited to participate. Participants completed two visits in a randomized, cross-over design with one week between visits. Subjects had 15 minutes to consume each breakfast. Blood glucose and appetite were assessed at baseline, 15, 30, 45, 60, and 120 minutes postprandial. Participants kept a 24-hour dietary record for the duration of each test day. No difference was found between NW and OW participants or breakfasts for postprandial appetite responses. AP had a significantly lower glucose response at 30 minutes compared with PP (−11.6%; 127 ± 4 versus 112 ± 4 mg/dL; *P* < 0.05) and a slower return to baseline. There was no difference in daily energy intake between breakfasts. These data suggest that protein source may influence postprandial glucose response without significantly impacting appetite response in breakfast consumers.

## 1. Introduction

Early adulthood is a vulnerable life stage for weight gain, especially among women. The average weight gain for women between the ages of twenty and thirty is 12–25 lbs [1]. Weight gain during early adulthood increases the risk of developing a number of chronic health conditions such as type 2 diabetes mellitus, osteoarthritis, and some cancers [2, 3]. For example, after the age of eighteen years, women are 1.9 times more likely to develop type 2 diabetes if body weight increased 10–16 pounds and were 2.7 times more likely to develop type 2 diabetes if body weight increased 16–22 pounds [1].

Breakfast is often cited as the most important meal of the day for children, but this is also true for adults. There are many benefits associated with eating a healthy breakfast including improved micronutrient intake, decreased incidence of overweight and obesity, and lower cholesterol levels [4–7]. Several studies, in both adults and children, have shown that individuals who eat breakfast tend to weigh less than those who omit breakfast as eating a healthy breakfast can reduce hunger throughout the day [8, 9]. Consuming more protein (20–30 g) at breakfast than found in the standard cereal-based breakfast (10–15 g) may increase subjective feeling of fullness and satiety throughout the day [10, 11] and decrease calorie intake at lunch [11]. In addition, overweight women consuming sources of protein for breakfast five times a week for eight weeks lost 65% more weight and reduced their waist circumference by 83% more than those participants eating a carbohydrate-based breakfast [10].

The use of high protein diets to reduce the amount of food consumed at the next meal is a strategy used to help maintain negative energy balance during weight loss or to maintain weight equilibrium [12]. Protein-based breakfasts positively affect postprandial blood glucose homeostasis, of which tighter control is strongly associated with a lower risk of type 2 diabetes, hypertension, and cardiovascular disease. Healthy participants as well as metabolically compromised individuals with type 2 diabetes both respond positively to high protein breakfasts, resulting in favorably altered biomarkers including reduced HbA1C%, postprandial glucose, postprandial insulin, and lower systolic blood pressure [13, 14].

Although several studies demonstrate positive effects of protein consumption at breakfast, very few have focused on the source or quality of the protein. Protein quality is important because although equal quantities of plant or animal protein may have the same caloric content, the digestibility and content of amino acids impact blood glucose regulation differently [15]. Therefore, the objective of this study is to determine if protein source (animal protein versus plant protein) at breakfast influences satiety and glucose response and decreases daily food intake.

## 2. Methods and Materials

### 2.1. Participants

**Figure 1:**
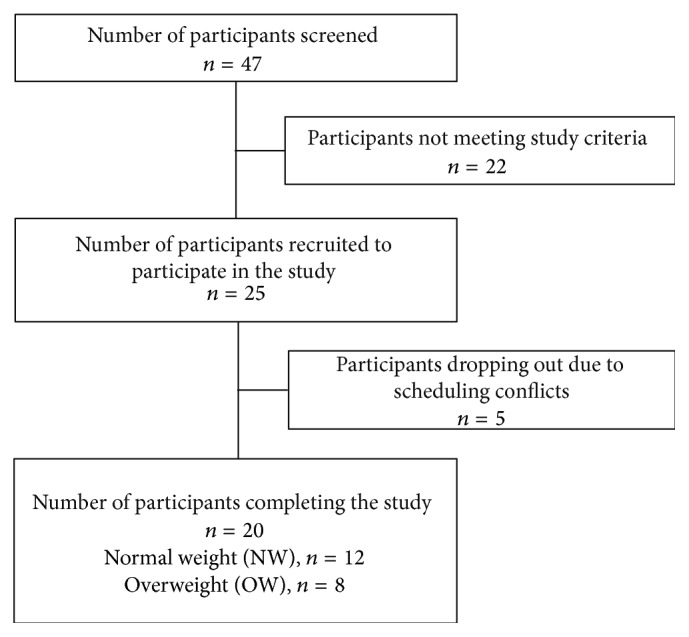
Flow diagram of the participant screening and selection process.

Female participants (*n* = 20; ages 18–36) were recruited using the university daily newsletter, social media, and word of mouth. Participants who were underweight (BMI ≤ 18.4), were smokers, were taking medication (with the exception of hormonal birth control), had food allergies and/or dietary restrictions (e.g., weight loss, vegetarian), disliked the foods served during the study, and/or had any known existing medical conditions that prevented them from eating the breakfasts were excluded from the study. Participants were recruited on a rolling basis and grouped based on their BMI score into normal weight (NW; BMI < 25; *n* = 12) or overweight (OW; BMI ≥ 25; *n* = 8) groups (Figure 1). A total of forty-seven women were screened and twenty-five participants started the study. Twenty-two of the women screened did not meet the study criteria. Twenty participants completed the study and were used in data analysis. Refer to Table 1 for participant characteristics. Females aged 18–36 were the focus of this study since this population is at a higher risk for weight gain [1] and there have been several papers published using the population that are focused on breakfast [16, 17]. Ethical approval for the study was obtained from the Office of Research Compliance Institutional Review Board of the University of Arkansas (Fayetteville, AR). Written consent was obtained from all participants prior to beginning the study.

### 2.2. Study Design

The study was conducted using a randomized, crossover design in which each subject received two different breakfasts, animal protein-based (AP) and plant protein-based (PP), with at least a one-week washout period between each test day and no more than 14 days between testing days. Participants were instructed to fast overnight and limit their physical activity prior to each study day. Upon arrival, baseline measurements of blood glucose and appetite were collected. Food items for each breakfast were portioned, weighed, and labeled appropriately for each subject. Participants were then given 15 minutes to consume the test breakfast. Participants were asked to rate the appearance and taste of the breakfast using a visual analog scale (VAS) [18]. Blood glucose and appetite were analyzed at 0, 15, 30, 45, 60, 90, and 120 minutes postprandial. In addition, participants were instructed to keep a 24-hour dietary food record for the remainder of each test day.

### 2.3. Test Breakfasts and Dietary Assessment

The nutritional composition for the test breakfasts is described in Table 2. The AP had 29% protein, 29% fat, and 42% carbohydrates. The PP breakfast consisted of 27% protein, 26% fat, and 47% carbohydrates. The AP breakfast consisted of one commercially available breakfast sandwich (Jimmy Dean Delights Turkey Sausage, Egg White, Cheese and English Muffin Breakfast Sandwich), 85 g plain, nonfat Greek yogurt, 6 almonds, and 85 g fresh blueberries. The PP breakfast contained 2 vegan sausage patties (76 g; MorningStar Farms, Kellogg's), 32.3 g of vegan country white bread (Rudi's), 1 slice of vegan American cheese (19 g; Go Veggie, Galaxy Nutritional Products), 85 g of blueberry soy yogurt (WholeSoy & Co.), and 28 g of fresh blueberries. Since we used commercially prepared products, we do not know the exact contribution of each protein source from each product. Participants were asked to record their food intake for the remainder of the test day using 24-hour dietary intake records. The participants were provided with detailed instructions and examples for completing the dietary intake records. The test breakfast composition and 24-hour dietary intake records were analyzed using the Genesis R&D diet analysis software package (Salem, OR).

### 2.4. Anthropometric Measurements

Body height was measured to the nearest 0.01 cm using a stadiometer (Detecto, St. Louis, MO) with participants barefoot, in the freestanding position. Body weight was measured in the fasting state with participants barefoot to the nearest 0.01 kg using calibrated balance scale (Detecto, St. Louis, MO). BMI was calculated as weight (kg) divided by height (m) squared.

### 2.5. Blood Glucose Measurement

Blood glucose samples were measured using the fingerstick method at 0, 15, 30, 45, 60, 90, and 120 minutes postprandial using a Lifescan One Touch UltraSmart System (New Brunswick, NJ). One blood sample per time point was collected in a capillary tube (Health Management Systems, Corp; Plano, TX). Samples were measured in duplicate from the sample collected in the capillary tube and the average was used in analysis [19, 20].

### 2.6. Appetite and Palatability Ratings

Participants were asked to rate their perceived hunger, fullness, desire for food, prospective food consumption, desire for something sweet, and desire for something savory using a 100 mm visual analog scale (VAS) [18]. The VAS is a validated questionnaire incorporating a 100 mm horizontal line scale with questions worded as “how strong is your feeling of” and end anchors of “not at all” to “extremely.” Taste and appearance of test breakfasts were collected using the same method.

### 2.7. Statistical Analysis

Summary statistics were calculated for all data (sample means and sample standard deviations). Net incremental area under the curve (niAUC) was calculated for appetite ratings and glucose values and was used in analyses [21]. Two-sample independent *t*-tests were used to determine initial differences between NW and OW participants and to analyze participant characteristics, breakfast appearance and palatability, and comparisons of niAUC between test breakfasts (AP versus PP). Twenty-four-hour energy and macronutrient intake were analyzed using one-factor analysis of variance (ANOVA). Two-factor, crossover, repeated measures analysis of variance (ANOVA) was used to examine significant differences between breakfast and weight groups over time for blood glucose and appetite ratings. The Bonferroni correction for multiple comparisons was applied when significance was observed within the analyses. Results are reported as means ± SEMs. All analyses were conducted using Prism GraphPad Software Version 6.0 (La Jolla, CA). *P* < 0.05 was considered statistically significant.

## 3. Results

### 3.1. Participant Characteristics and Compliance

The physical characteristics of the participants are presented in Table 1. There was no difference in age or height between the NW and OW groups. Body weight and BMI were higher in the OW group (*P* < 0.05).

### 3.2. Appetite and Palatability Responses

**Figure 2:**
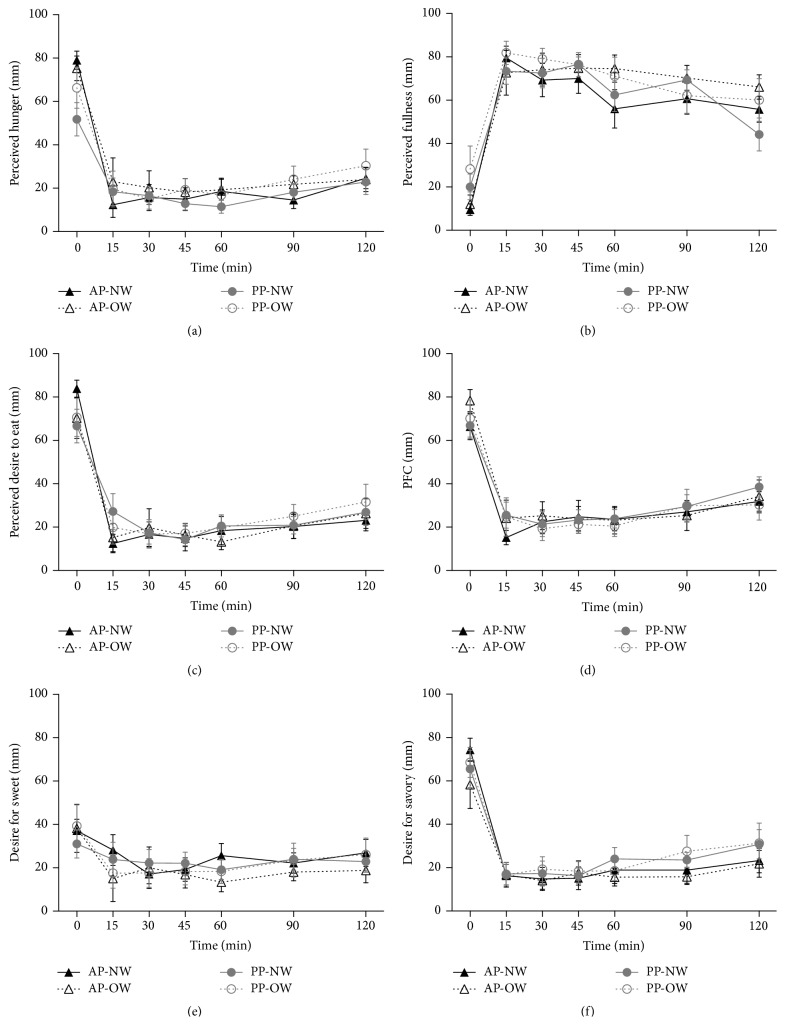
Appetite responses following test breakfasts. Values expressed as means ± SEM. Data are depicted as appetite rating over time per weight group and breakfast type and net incremental area under the curve (niAUC). (a) Perceived hunger. (b) Perceived fullness. (c) Perceived desire to eat. (d) Prospective food consumption. (e) Desire for something sweet. (f) Desire for something savory. AP: animal protein; NW: normal weight; OW: overweight; PP: plant protein.

The results for perceived hunger, fullness, desire to eat, prospective food consumption, and food cravings are presented in Figure 2. There was no difference in appetite ratings or food cravings between NW and OW groups or between AP and PP breakfasts. However, there was an effect of time on both appetite and food cravings for both group and breakfast (*P* < 0.0001).

The perceived taste and appearance responses to each breakfast were measured immediately following breakfast consumption. There was no difference in taste between AP or PP breakfasts (Table 2). Participants preferred the appearance of the AP versus the PP breakfast (*P* < 0.05).

### 3.3. Blood Glucose Response

**Figure 3:**
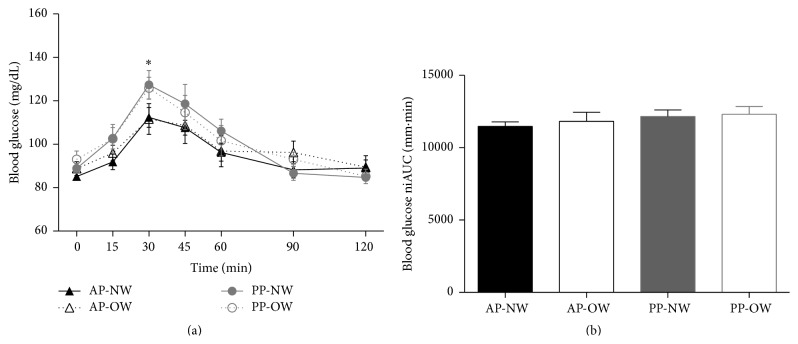
Glucose response to the test breakfasts. (a) Glucose response to the test breakfasts over time. (b) Glucose net incremental area under the curve (niAUC). Values expressed as means ± SEM. *∗* indicates that blood glucose values for AP were significantly different than PP (*P* < 0.05). AP: animal protein; NW: normal weight; OW: overweight; PP: plant protein.

The results for postprandial glucose response are presented in the line graphs (individual time points) and bar graphs (niAUC) in Figure 3. Overall, there was an effect of time on postprandial blood glucose response (*P* < 0.0001), with no effect of diet or weight group over time. Postprandial blood glucose was higher at 30 min following with PP breakfast compared to the AP breakfast, 126.8 ± 4.4 mg/dL versus 112.1 ± 3.9 mg/dL, respectively (*P* < 0.05). Participants had a lower percent change in blood glucose response from the postprandial peak at 30 min to 120 min postprandial following the AP breakfast versus the PP breakfast (−26.9 ± 4.3% and −46.5 ± 4.9%, resp.; *P* < 0.01).

### 3.4. 24-Hour Food Intake Assessment

Nutrient composition of the 24-hour food intake records is shown in Table 3. Overall, there was no difference in 24-hour nutritional intake between weight groups or breakfast type. However, there was a trend for participants to have a higher caloric intake following the AP breakfast compared to the PP breakfast (*P* = 0.09). In general, the OW group ate an additional 133 kcal more than NW group. The OW group consumed on average 44% of kcals from carbohydrate, 38% of kcals from fat, and 17% of kcals from protein after each test breakfast, while the NW group consumed on average 53% of kcals from carbohydrate, 36% of kcals from fat, and 21% of kcals from protein.

## 4. Discussion

This is one of the first studies to examine the effect of complete meals comparing plant protein and animal protein sources, on postprandial appetite and glucose response in NW and OW females. The present study suggests protein source within the context of a higher protein meal exhibits no difference in appetite response or total nutritional intake; however, protein source could play a role in regulating postprandial blood glucose levels by decreasing the postprandial peak in blood glucose levels.

No difference in postprandial appetite response between AP or PP was detected; however, these results are consistent with several studies in current literature that have tested isolated proteins that were not part of a complete meal. Several studies have compared the effect of protein source on appetite within a mixed meal [22–24], demonstrating equal appetite responses to plant and animal proteins within higher protein meals (>22% protein). When whey protein was compared to casein and soy at 10% energy of a test breakfast, whey exhibited a greater satiating response; however, this difference diminished when the protein level was increased to 25% energy of a test breakfast, which is similar to the higher protein breakfast composition used in this study [22]. Another study examined beef versus soy within a mixed meal and found no difference in hunger or fullness responses over seven hours [24]. The similar effect of protein sources on appetite response within a high protein diet may be attributed to an overall increased consumption of amino acids [25, 26].

Furthermore, fiber is known to influence appetite response [27]. Although PP breakfast had a slightly higher fiber content (1 g) compared to AP, there is evidence that fiber quantity may have little impact on satiety within a high protein diet. One study demonstrated that when mixed meals, matching in protein content with differing fiber amounts, were ingested, there was no difference found hunger or fullness area under the curve analysis [28] suggesting that protein quantity may influence satiety to a greater extent than fiber content. However, additional research needs to be explored comparing high protein/fiber diets and their effect on appetite.

An increase in protein intake throughout the day, starting with breakfast, may help an individual to feel more satisfied and respond to neural signals of satiety and blood glucose regulation [29]. Though not significant, OW participants consumed fewer calories following the AP breakfast. In general, OW participants consumed less protein and consumed more calories compared to NW participants over the 24-hour test period. The underlying mechanism is still unknown, but high protein diets appear to spontaneously reduce food intake in individuals which could be attributed to satiating effect of protein [30].

Despite there being no significant differences in glucose response between breakfasts or weight groups over the 120 min postprandial period (niAUC), there was a trend for a more stable postprandial glucose response following AP breakfast for both NW and OW groups. The control of postprandial glucose levels is important for HbA1C% levels and diabetes risk [31, 32]. Both eucaloric and hypocaloric diets with increased protein lead to more stable postprandial glucose levels with lesser peak excursions and incremental area under the curve [33–36]. The higher postprandial glucose levels for both NW and OW following the PP breakfast could be attributed to the disparity in breakfast carbohydrate content or differing amino acid profiles of the test breakfasts. It has been observed that healthy individuals and those with higher postprandial glucose levels may do better with a high animal protein-based breakfast compared to a lower protein, carbohydrate-based breakfast [17]. Another possibility is that the lower blood glucose observed, following the AP breakfast, could be due to an increase in insulin production; however, insulin response was not measured in this study and needs to be further explored.

### 4.1. Limitations

The first limitation of this study is the short postprandial data collection period following breakfast consumption. Two hours postprandial may not be enough time to fully capture the postprandial appetite and glucose response, as meals are generally four to five hours apart and initiated by habit or hunger [37]. Many studies take postprandial measurements for four hours or longer following the test meal to ensure that appetite responses and metabolic measurements (e.g., glucose) return to baseline [16, 24]. Therefore, we may not have captured the entire postprandial breakfast response. Since there were no differences in postprandial appetite responses niAUC, we do not think measuring over a longer period would change our results. Additionally, the discrepancy in caloric and carbohydrate values and fiber content of the test breakfasts may have contributed to the differences observed in postprandial glucose response. The AP breakfast had lower postprandial glucose response at 30 min, which could be due to the lower carbohydrate and fiber content of this breakfast. However, since our conclusions are consistent with current literature, they do not warrant dismissal [22, 26, 38]. Finally, blood glucose was measured via fingerstick, not via intravenous blood draw, which limited the number of postprandial analyses conducted.

## 5. Conclusions

There was no difference in postprandial appetite response or 24-hour food intake after consumption of breakfasts higher in protein with differing protein sources, AP versus PP, in either NW or OW women. However, consumption of PP generated a higher postprandial glucose peak compared to AP. Taken together, these data suggest that protein source, as part of breakfast higher in protein, does not differentially affect appetite response but may differentially affect postprandial metabolism.

## Figures and Tables

**Table 1:** Participant characteristics^1^.

Characteristics	NW	OW
Participants (*n*)	8	12
Age (y)	25 ± 1^a^	25 ± 1^a^
Weight (kg)	61.3 ± 2.1^a^	87.8 ± 7.8^b^
Height (m)	1.66 ± 1.2^a^	1.65 ± 1.8^a^
BMI (kg/m^2^)	22.2 ± 0.6^a^	31.9 ± 2.7^b^
Ethnicity		
Asian	2	0
Caucasian	7	6
Indian	2	1
Latina	1	1

^1^Age, weight, height, and BMI are expressed as means ± SEM. NW: normal weight participants; OW: overweight participants. Means in a row without a common letter are significantly different (*P* < 0.05).

**Table 2:** Dietary characteristics of test breakfasts.

	Animal protein (AP) breakfast	Plant protein (PP) breakfast
Total kcal	368	387
Protein (g)	27	26
Fat (g)	12	11
Carbohydrate (g)	38	46
Fiber (g)	4	5
Breakfast appearance, mm^1^	74.8 ± 3.6^a^	63.6 ± 3.5^b^
Breakfast palatability, mm^1^	73.1 ± 3.5^a^	65.9 ± 3.8^a^

^1^Values are expressed as means ± SEM, *n* = 20. CHO: carbohydrate-based breakfast; PRO: protein-based breakfast. Means in a row without a common letter are significantly different (*P* < 0.05).

**Table 3:** Energy and macronutrient content of 24-hour food intake.

	AP-NW	AP-OW	PP-NW	PP-OW
Energy (kcal)	2327 ± 141	2417 ± 251	2041 ± 161	2218 ± 269
Carbohydrate (g)	271 ± 13.3	275.6 ± 22.9	308.18 ± 55.6	237.6 ± 35.3
Fat (g)	93.5 ± 11.4	100.4 ± 13.7	83.1 ± 19.8	95.6 ± 13.7
Protein (g)	123.1 ± 20.9	107.3 ± 20	107.4 ± 10.5	93.4 ± 14.1

^1^Values are expressed as means ± SEM. AP: animal protein; NW: normal weight; OW: overweight; PP: plant protein.

## Abbreviations

PRO: Protein
CHO: Carbohydrate
NW: Normal weight
OW: Overweight
AP: Animal protein-based breakfast
PP: Plant protein-based breakfast
VAS: Visual analog scale
BMI: Body mass index.
